# Blood Alcohol Concentration in Finnish Suicide Deaths and Associated Risk Factors, 2016–2024

**DOI:** 10.1111/acps.70100

**Published:** 2026-04-17

**Authors:** Tristan Pokornyi, Marjut Grainger, Timo Partonen

**Affiliations:** ^1^ Finnish Institute for Health and Welfare (THL) Department of Healthcare and Social Welfare, Promotional and Preventive Work Unit, Mental Health Team Helsinki Finland; ^2^ Faculty of Social Sciences, Kauppi Campus Tampere University Tampere Finland

**Keywords:** forensic medicine, population, psychiatry, risk, suicidal behaviour

## Abstract

**Aims:**

There is limited research examining the relationship between blood alcohol concentration (BAC) and other risk factors among suicide deaths in Finland. Our study aimed to investigate the relationship of an elevated (more than zero) blood alcohol concentration with medical history, including sociodemographic characteristics and disease diagnoses.

**Methods:**

Data was collected from suicide deaths in Finland from 2016 to 2024 and verified by official cause‐of‐death investigations, which included forensic autopsy, toxicology results, and other key information from death certificates and national healthcare registries. A condition recorded in both the death certificate (autopsy) and healthcare register was considered as confirmed diagnosis. BAC, as expressed as a percentage, was grouped into three categories: nil (BAC = 0.000), low‐to‐medium (BAC = 0.010%–0.099%), and high (BAC = 0.100%–0.500%). Descriptive statistics, correlation, and stepwise logistic regression to estimate odds ratios (ORs) for BAC categories were undertaken.

**Results:**

The number of suicide deaths from 2016 to 2024 in Finland was *n* = 6892, with 5183 men (75.2%) and 1709 women (24.8%). BAC reports were available for *n* = 6835. Independent factors associated with a low‐to‐medium or high BAC at death were alcohol use (ORs: 41.80–275.27), longer than 1 day since last healthcare visit (ORs: 1.54–2.53), previous suicide attempt(s) (ORs: 1.42–1.65), and female gender (only for high BAC, OR: 1.30). There were reduced odds for schizophrenia spectrum diagnosis (OR: 0.17), bipolar disorder (OR: 0.29), age groups 10–19 (ORs: 0.30–0.65), 80 and over (ORs: 0.15–0.39), and 70–79 years (only for high BAC, OR: 0.43).

**Conclusion:**

Our findings show that individuals with documented habitual alcohol use, female gender, previous suicide attempts, and less frequent healthcare visits had higher odds of intoxication at suicide. Meanwhile, being aged under 20, over 70, or diagnosed with schizophrenia or bipolar disorders reduced the odds. The results highlight the need for proactive healthcare engagement and integrated alcohol‐use interventions in suicide prevention strategies.

## Significant Outcomes

- Documented habitual alcohol use, infrequent healthcare contact, previous suicide attempt(s), and female gender were key factors associated with increased odds of high blood alcohol concentration at suicide.
- A confirmed diagnosis of schizophrenia, bipolar disorder, being aged under 20 or over 70 were key factors associated with reduced odds of high BAC at suicide.
- Our study findings indicate that increased healthcare engagement and alcohol‐use interventions would benefit individuals at risk of suicide.

## 1 Introduction

Suicide is one of the leading causes of death globally, with between 700,000 and 800,000 deaths per year, almost 10 per 100,000 population, with a significant public health burden [1, 2]. In Finland, suicide deaths have had a decreasing trend since 1990, although they are still higher than the European Union average [3]. Well‐developed community mental healthcare and outpatient services have contributed to this decreasing trend. The most common methods of suicide have been hanging, self‐poisoning, substance overdose, and firearms [3]. Suicide is closely linked with mental disorders and conditions such as severe depression, bipolar disorder, schizophrenia, post‐traumatic stress disorder, alcohol and substance abuse [4, 5, 6]. Socio‐demographic factors contributing to suicide risk include unemployment, poverty, age, access to healthcare, and male gender [2]. Furthermore, a previous suicide attempt is the leading risk factor for suicide deaths [7]. Alcohol use is commonly observed in individuals with mental disorders and suicidal behaviour. Around one‐third of people who die by suicide have alcohol or drugs present in their system at the time of death [8].

### 1.1 Terminology

Our definition of suicidal behaviour in this study is consistent with academic consensus. Suicide is someone intentionally ending their own life, and suicidal behaviour includes three categories, of which any or all can be included: ideation, plan, and attempt [9].

### 1.2 Suicidal Behaviour and Mental Health Disorders

Psychiatric disorders, both diagnosed and unreported, are present in most suicide attempt cases. Over 60% of individuals who died by suicide had a mental disorder [2]. A meta‐analysis by Too et al. [5] found the risk for suicide among individuals with a mental disorder increased eightfold. The odds ratio was 13.2 for psychotic disorders, 12.3 for mood disorders, 8.1 for personality disorders, 4.4 for substance use disorders, and 4.1 for anxiety disorders. Financial hardship, interpersonal difficulties, and physical health concerns are also risk factors, and many suicide cases were not diagnosed with a mental health disorder. There is evidence that care‐seeking behaviour is reduced in individuals who attempt or complete suicide; according to a study by Dahaban et al. [10], 68% of suicide deaths had no contact with mental health services in the year before.

### 1.3 Suicidal Behaviour and Alcohol Use

There is a significant body of research regarding suicidal behaviour and alcohol use. Alcohol consumption and high blood alcohol concentrations (BACs) can influence suicide through disinhibition, impulsivity and impaired decision‐making, and can also ease distress when individuals engage in suicidal behaviour [11]. Alcohol intoxication, along with drug overdose, is a specific method of suicide in some cases. Heavy alcohol consumers have been found to have a fivefold higher risk of suicide than social drinkers, and about 40% of patients seeking treatment for alcohol dependence report at least one lifetime suicide attempt [11]. Meanwhile, a psychological autopsy study found that 68% of males and 29% of females who died by suicide met the criteria for alcohol dependence [12]. Suicide risk increases with the level of alcohol consumed in a dose‐dependent effect.

Heavy alcohol use is frequently linked to sadness in general, although a causal relationship has not been established. Schuckit et al. [13] found in a longitudinal study (*n* = 397) that of those who developed alcohol use disorders, 31% of depressive episodes were substance related. Conversely, those who developed independent depressive episodes did not have an increased rate of alcohol use disorders. Although often occurring together, alcohol use disorders and depressive disorders likely involve different mechanisms and should be assessed separately in suicide cases. Agrawal et al. [14] identified that alcohol and drug use, misuse, and abuse tends to co‐occur with suicidal thoughts and behaviours. While ideation had 0.71–0.77 odds of subsequent substance use, attempt was associated with 1.44–1.61 odds of later alcohol and substance dependence. This indicates that suicidal attempt, but not necessarily thoughts, is associated with a greater likelihood of subsequent alcohol and drug use disorders.

Individuals with alcohol dependence have 60–120 times higher suicide risk than those without psychiatric diagnosis according to Sher [15]. In a study of 6744 suicide cases available in an Australian registry, Kõlves et al. [16] identified that 32% of cases had an elevated BAC of > 0.05%, with slightly higher prevalence in males compared to females. Younger and middle‐aged groups of 25–44 years were most likely to have an elevated BAC at time of death, and older adults 65 and over were least likely. Comorbidity of psychiatric disorders was less likely in elevated BAC deaths, with odds from 0.34 to 0.74, while substance use disorders had increased odds of 2.05. Relationship and interpersonal conflict also had increased odds. This suggests that suicide deaths involving alcohol differ in key risk factors from those driven by psychiatric diagnoses.

### 1.4 Socio‐Demographic Risk Factors for Suicidal Behaviour

There are several sociodemographic risk factors for suicidal behaviour. Men are overrepresented as suicide victims compared to women, with a ratio of approximately 1.7:1 worldwide [2]. A systematic review by Cano‐Montalban and Quevedo‐Blasco [17] found that though men and the elderly died by suicide more frequently, there are more suicide attempts among women. In the same review, unemployment, rural life, unmarried relationship status, and low education were highlighted as risk factors. Notably, older men had higher odds of suicide death, while for younger women there was increased ideation, planning, and attempts. Berkelmans et al. [18] examined a training set of 5854 suicide deaths and 596,416 control cases in the Netherlands with similar findings; but there were increased odds for death by suicide in middle‐aged people, individuals with low income, and those living alone. There were reduced odds for individuals that were highly educated, from a non‐western immigrant background, and living with a partner. Homeless people and sexual and gender minorities have been reported by many studies to have increased odds; however, the data on suicidal behaviour in migrant and asylum seeker groups varies across studies [2].

## 2 Study Aims

Our study aimed to investigate the relationship between an elevated (more than zero) BAC and medical history, including sociodemographic characteristics and disease diagnoses, at time of death among 6892 suicide cases in Finland from 2016 to 2024. Understanding BAC‐related risk factors in suicide deaths can inform effective prevention strategies in psychiatric and primary care settings.

Our research question is whether there is an association between an elevated BAC and the medical history of Finnish suicide cases, including disease diagnoses and sociodemographic characteristics.

Our Hypothesis 1 is that specified diagnoses including depression, schizophrenia, and bipolar disorder will have a statistically significant negative association with elevated BAC at time of suicide.

Our Hypothesis 2 is that male gender, documented habitual alcohol use, a greater duration of time since previous visit to healthcare services, and previous suicide attempts will have a statistically significant positive association with elevated BAC at time of suicide.

## 3 Methods

The data comprised all the deaths from suicide (*n* = 6892), verified by official cause‐of‐death investigations, which included forensic autopsy, analysis of forensic toxicology samples and other key information from death certificates between January 1, 2016 and December 31, 2024 in Finland. BAC reports were available for 6835 suicide deaths. The data were obtained from the Forensic Medicine Unit of the Finnish Institute for Health and Welfare (THL), the legal authority in charge of death investigations in Finland, prior to inclusion in this study. Healthcare register data on all the suicide patients was collected from the two national databases (Care Register for Health Care, Register of Primary Health Care visits), including previous diagnoses and visits to healthcare services. The final data were then compiled from the database of THL. Protected personal data was anonymised. The population of Finland between 2016 to 2024 increased slightly, from 5.495 million in 2016 to 5.637 million in 2024.

Read‐outs of BAC (in g/L) were taken from the toxicology component. The Toxicological Laboratory at the Department of Forensic Medicine, University of Helsinki, is part of the Forensic Chemistry unit of the Finnish Institute for Health and Welfare, conducts postmortem toxicological analyses, and serves as the centralised, national expert body (Testing Laboratory No. T115) for forensic toxicology, alcohol, and drug testing in Finland. It uses dual‐column head‐space gas chromatography to measure BAC and conducts postmortem toxicological analysis for all medico‐legal autopsies in Finland during the study period. Its analytical methods have been accredited since 1997 by the Centre for Metrology and Accreditation according to SFS‐EN 45001 ISO/IEC Guide 25. The BAC results were then expressed as a percentage and categorised into nil, low‐to‐medium (0.010%–0.099%) and high (0.100%–0.500%). This was done in order to have sufficient group sizes to perform effective statistical analyses and measure dose‐dependent effects between groups.

The authors assert that all the procedures contributing to this work comply with the ethical standards of the relevant national and institutional committees on human experimentation, as well as with the Declaration of Helsinki as adopted by the World Medical Association in 1964 and amended thereafter. All the procedures involving human participants were approved (Dnro THL/2010/6.02.00/2018) by the institutional review board (IRB 00007085) of THL (FWA 00014588) on 20 November 2018.

### 3.1 Statistical Analysis

First, descriptive statistical analysis on the population was undertaken. Second, we performed a Spearman correlation analysis of only death certificate (autopsy) diagnoses to find disease diagnoses that were statistically significant and with the highest correlation coefficients in relation to elevated BAC. Spearman's correlation analysis was chosen due to the categorical nature of the variables, and to initially explore possible associations. Registry and death certificate (autopsy) diagnoses were then re‐coded into combined variables to assist in the accuracy of the analysis, with 1 = register diagnosis, 2 = autopsy diagnosis, 3 = combined diagnosis, and 4 = no diagnosis. Several sociodemographic variables measured by scale were re‐coded into categorical variables.

A multinomial logistic regression with nil BAC as the reference category was performed with the forward stepwise method. The dependent variable in the logistic regression was BAC category, in which 0 = nil, 1 = 0.010%–0.099% (low‐to‐medium), and 2 = 0.100%–0.500% (high). Sociodemographic, medical history, and theoretically relevant diagnostic variables were chosen as independent variables. Those with elevated correlation coefficients were selected as forced entry terms. The remainder were forward stepwise terms. Elevated odds ratios (ORs) in sociodemographic subcategories and medical variables with combined diagnosis were the focus of the results and discussion. Analysis of the data was done by IBM SPSS Statistics, version 29.0 (International Business Machines Corporation, Armonk, NY, USA).

## 4 Results

**FIGURE 1:**
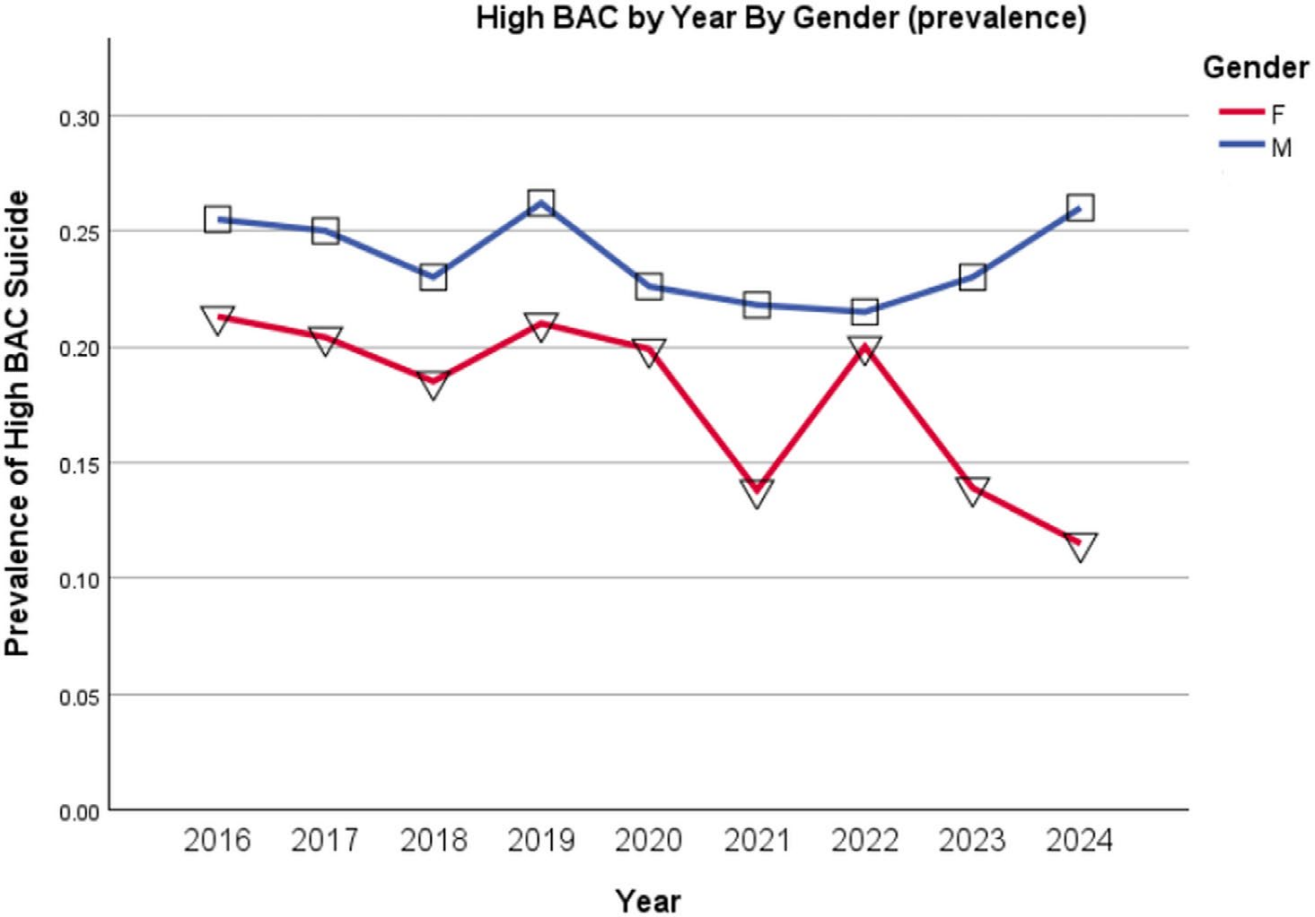
High BAC by suicide year by gender prevalence in Finland (proportion).

Of 6892 suicide deaths in Finland from 2016 to 2024 there were 5183 men (75.2%) and 1709 women (24.8%). Most of the studied population (63.6%) had nil BAC at death, 13.5% had a low‐to‐medium BAC of 0.010%–0.099% and 22.2% had a high BAC of 0.100% and above (further details in Table 1 and Figure 1). There was a slight decrease of suicide deaths on average between 2016 and 2024 (see Appendix A). Suicide by region reflected the overall population density of Finland, with most cases in more densely populated regions. The median age at death was 47 for both men and women. There was a slight decrease in the prevalence of high‐BAC suicide deaths in women from 2016 to 2024.

**TABLE 1:** Demographic and medical history of studied population.

Variable	Category	Frequency	Percent
Gender	Male	5183	75.2
Gender	Female	1709	24.8
Age at death	10–19	331	4.8
Age at death	20–29	1143	16.6
Age at death	30–39	1149	16.7
Age at death	40–49	1056	15.3
Age at death	50–59	1108	16.1
Age at death	60–69	977	14.2
Age at death	70–79	688	10
Age at death	80 and over	440	6.4
Earlier suicide attempts	None	5413	78.5
Earlier suicide attempts	One	494	7.2
Earlier suicide attempts	Two or more	985	14.3
Last healthcare visit	1–7 days	1795	26
Last healthcare visit	Past month	1372	19.9
Last healthcare visit	1–6 months ago	1267	18.4
Last healthcare visit	6–12 months ago	332	4.8
Last healthcare visit	1–2 years ago	251	3.6
Last healthcare visit	More than 2 years ago	232	3.4
Last healthcare visit	Within a day or unspecified	1558	22.6
Blood alcohol concentration	Nil BAC 0.000	4380	63.6
Blood alcohol concentration	Low‐to‐medium BAC 0.010–0.099	927	13.5
Blood alcohol concentration	High BAC 0.100–0.500	1528	22.2
Citizenship	Foreigner	89	1.3
Citizenship	Finnish citizen	6803	98.7

In our Spearman's correlation analysis, of 25 reported disease variables, 10 were statistically significant at the *p* < 0.05 level, and 6 were statistically significant at the *p* < 0.01 level. These six were cancer, respiratory diseases, alcoholic diseases, alcohol use, schizophrenia group psychosis, and bipolar disorder. Each of these had either a low positive or low negative Spearman correlation coefficient, ranging from −0.102 to +0.046, except for alcohol use, which had a high positive correlation coefficient of +0.723. This indicates that death certificate diagnoses of the reported conditions had weak or non‐existent associations with elevated‐BAC suicide deaths, with the exception of alcohol use. Details on the correlation analysis can be found in Table 2.

**TABLE 2:** Spearman's rho correlation of death certificate or autopsy confirmed diseases in relation to blood alcohol concentration (BAC) level at death in Finnish suicide cases from 2016 to 2024.

Contributing disease on death certificate correlated with BAC	Spearman *ρ*	*Sig.*
Cancer C00–D48	**−0.049**	< 0.001**
Diabetes: E10–E14	−0.017	0.160
Other endocrine diseases excluding diabetes	0.008	0.485
Circulatory diseases: I00–I99	0.006	0.632
Diseases of the musculoskeletal and connective tissue	−0.004	0.757
Respiratory diseases: J00–J99	**−0.037**	**0**.002**
Alcoholic diseases	**0.046**	< 0.001**
Dementia: F00‐03, G30, G31.0	**−0.024**	**0**.**045**
Alcohol use: F10*	**0.723**	< 0.001**
Drug use: F11*–F19*	0.012	0.304
Schizophrenia group psychosis: F20–29	**−0.102**	< 0.001**
Bipolar disorder: F30–31, F34.0	**−0.041**	< 0.001**
Major and persistent depression: F32	0.006	0.627
Other mood disorder: F34.8, F34.9, F38, F39	−0.016	0.187
Anxiety disorders: F40–F43	**−0.029**	**0**.017*
Other anxiety related disorders F40: F44–49	**−0.028**	**0**.022*
Eating disorders: F50	−0.009	0.469
Perinatal mental health disorders: F53	—	—
Other eating disorders F50: F51, F52, F54–59	**−0.029**	**0**.016*
Personality disorders: F60–F62, F69	−0.018	0.140
Other personality disorders F60: F63–F68	0.002	0.838
Intellectual disability: F70–79	−0.009	0.469
Mental developmental disorders F80–89	−0.007	0.562
Typically begin in childhood or adolescence	−0.004	0.749
unspecified mental health disorder F9	−0.008	0.516

*Note:* * statistically significant at the *p* < 0.05 level ** statistically significant at the *p* < 0.01 level (in bold).

### 4.1 Multinomial Logistic Regression

**FIGURE 2:**
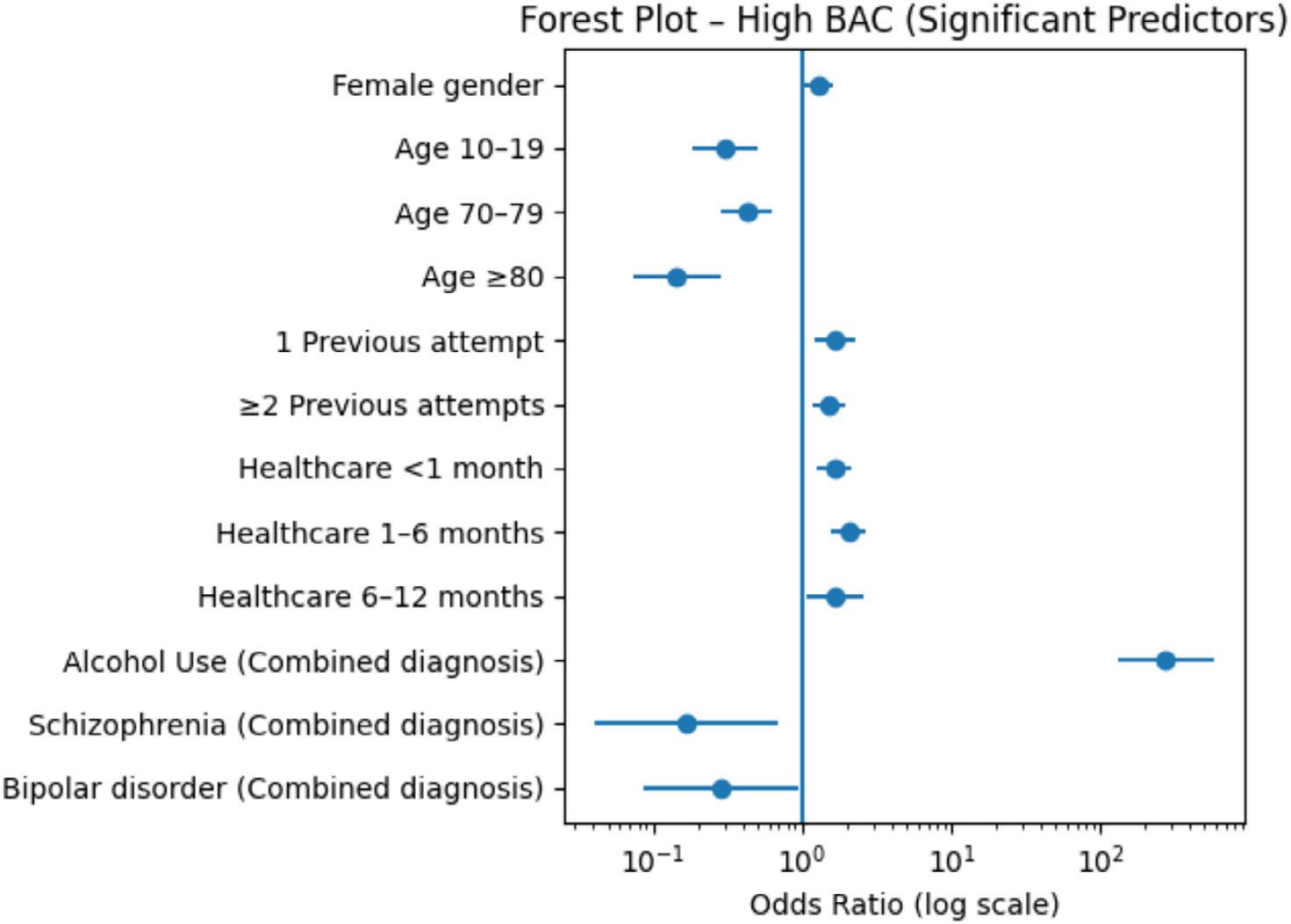
Statistically significant predictors of high BAC at suicide (odds ratio, log odds scale).

**FIGURE 3:**
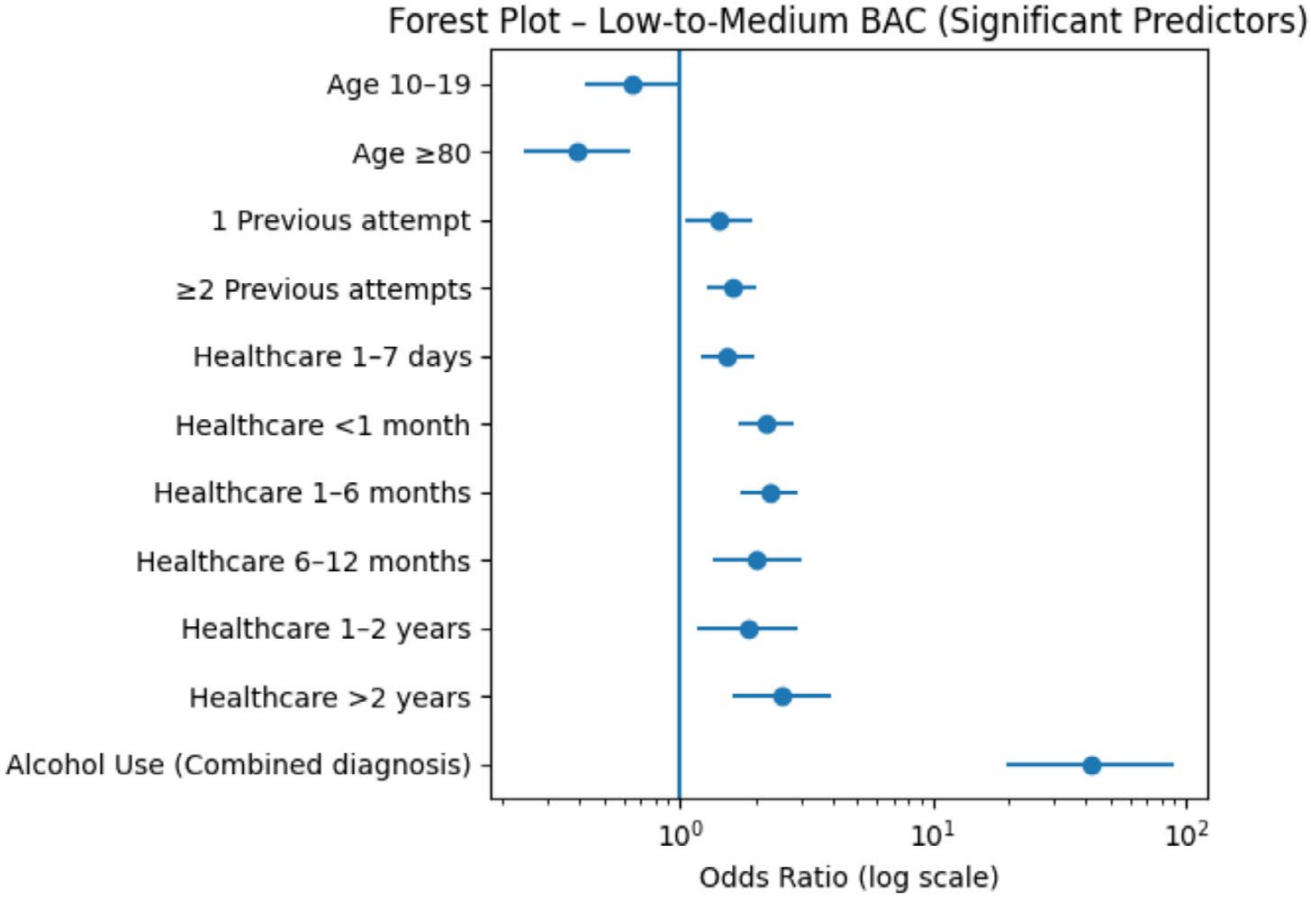
Statistically significant predictors of low‐to‐medium BAC at suicide (odds ratio, log odds scale).

Table 3 shows the odds ratios (ORs) of included sociodemographic and medical characteristics, with nil BAC as the reference category. The ORs for diagnosis were based on the combined diagnosis on both the death certificate (autopsy) and healthcare register. Figures 2 and 3 show the ORs of statistically significant factors. Statistical significance was set at *p* < 0.05.

**TABLE 3:** Odds ratio of each sociodemographic characteristic and medical condition included in our study's multinomial logistic regression analysis.

	Sig.	Odds ratio (Exp(*B*))	95% confidence interval lower bound	95% CI upper bound
*Low‐to‐medium BAC 0.01–0.09*				
Gender: female	0.945	1.007	0.833	1.217
Gender: male (REF)				
Age at death: 10–19	**0.047**	**0.647**	**0.421**	**0.994**
Age at death: 20–29	0.699	1.055	0.804	1.384
Age at death: 30–39	0.962	1.007	0.765	1.324
Age at death: 40–49 (REF)				
Age at death: 50–59	0.749	1.047	0.791	1.385
Age at death: 60–69	0.327	0.863	0.642	1.159
Age at death: 70–79	0.381	0.864	0.623	1.199
Age at death: 80 and above	**< 0.001**	**0.390**	**0.241**	**0.631**
Earlier attempts = 1	**0.021**	**1.422**	**1.055**	**1.917**
Earlier attempts = 2 or more	**< 0.001**	**1.604**	**1.283**	**2.005**
Earlier attempts = none (REF)				
Last visit to healthcare = 1–7 days ago	**0.001**	**1.540**	**1.205**	**1.968**
Last visit to healthcare = more than a week but less than a month	**< 0.001**	**2.176**	**1.692**	**2.799**
Last visit to healthcare = 1–6 months ago	**< 0.001**	**2.254**	**1.735**	**2.930**
Last visit to healthcare = 6–12 months ago	**0.001**	**2.009**	**1.340**	**3.014**
Last visit to healthcare = 1–2 years ago	**0.008**	**1.862**	**1.179**	**2.942**
Last visit to healthcare = More than 2 years ago	**< 0.001**	**2.529**	**1.625**	**3.936**
Last visit to healthcare = within past day (REF)				
Citizenship = foreigner	0.169	0.344	0.075	1.576
Citizenship = Finnish citizen (REF)				
Cancer diagnosis = both	0.667	1.360	0.335	5.529
Cancer diagnosis = none (REF)				
Alcoholic disease diagnosis = both	0.264	3.417	0.396	29.456
Alcoholic disease diagnosis = none (REF)				
Alcohol use = both	**< 0.001**	**41.799**	**19.361**	**90.242**
Alcohol use = none (REF)				
Drug use = both	0.079	1.611	0.946	2.744
Drug use = none (REF)				
Schizophrenia = both	0.872	1.064	0.501	2.262
Schizophrenia = none (REF)				
Bipolar disorder = both	0.563	0.755	0.292	1.955
Bipolar disorder = none (REF)				
Major depression = both	0.323	1.197	0.838	1.711
Major depression = none (REF)				
Respiratory disease diagnosis = both	^a^	^a^	^a^	^a^
Respiratory disease diagnosis = none (REF)				
Circulatory diseases diagnosis = both	0.526	1.401	0.494	3.972
Circulatory diseases diagnosis = none (REF)				
*High BAC (0.100–0.500)*				
Gender: female	**0.017**	**1.297**	**1.047**	**1.605**
Gender: male (REF)				
Age at death: 10–19	**< 0.001**	**0.306**	**0.185**	**0.507**
Age at death: 20–29	0.235	0.835	0.620	1.124
Age at death: 30–39	0.519	0.907	0.674	1.220
Age at death: 40–49 (REF)				
Age at death: 50–59	0.282	1.173	0.877	1.570
Age at death: 60–69	0.079	0.753	0.550	1.033
Age at death: 70–79	**< 0.001**	**0.427**	**0.289**	**0.631**
Age at death: 80 and above	**< 0.001**	**0.145**	**0.074**	**0.283**
Earlier attempts = 1	**0.002**	**1.650**	**1.196**	**2.278**
Earlier attempts = 2 or more	**0.001**	**1.526**	**1.184**	**1.967**
Earlier attempts = none (REF)				
Last visit to healthcare = 1–7 days ago	0.177	1.204	0.920	1.577
Last visit to healthcare = more than a week but less than a month	**< 0.001**	**1.646**	**1.250**	**2.167**
Last visit to healthcare = 1–6 months ago	**< 0.001**	**2.041**	**1.548**	**2.691**
Last visit to healthcare = 6–12 months ago	**0.021**	**1.665**	**1.079**	**2.567**
Last visit to healthcare = 1–2 years ago	0.141	1.443	0.886	2.351
Last visit to healthcare = More than 2 years ago	0.382	1.255	0.755	2.085
Last visit to healthcare = within past day (REF)				
Citizenship = foreigner	0.880	1.095	0.336	3.574
Citizenship = Finnish citizen (REF)				
Cancer diagnosis = both	0.320	0.283	0.023	3.410
Cancer diagnosis = none (REF)				
Alcoholic disease diagnosis = both	0.355	2.867	0.308	26.723
Alcoholic disease diagnosis = none (REF)				
Alcohol use = both	**< 0.001**	**275.270**	**131.999**	**574.045**
Alcohol use = none (REF)				
Drug use = both	0.945	1.025	0.505	2.081
Drug use = none (REF)				
Schizophrenia = both	**0.013**	**0.168**	**0.041**	**0.690**
Schizophrenia = none (REF)				
Bipolar disorder = both	**0.040**	**0.285**	**0.086**	**0.943**
Bipolar disorder = none (REF)				
Major depression = both	0.856	1.038	0.692	1.559
Major depression = none (REF)				
Respiratory disease diagnosis = both	^a^	^a^	^a^	^a^
Respiratory disease diagnosis = none (REF)				
Circulatory diseases diagnosis = both	0.622	1.335	0.423	4.214
Circulatory diseases diagnosis = none (REF)				

^a^ Not estimable—predictors with sparse data or unstable estimates were excluded from the main results table (see appendices for full output).

### 4.2 Sociodemographic Characteristics

Female gender was associated with a higher BAC at suicide with a statistically significant odds ratio (OR) of 1.30 for high BAC, with the result not reaching significance for low‐to‐medium BAC. There were reduced odds for age‐groups of 10–19 (OR = 0.30), 70–79 (OR = 0.43), and 80 or over (OR = 0.15) for high BAC compared to the reference group of 40–49. Age‐groups 10–19 (OR = 0.65) and 80 or over (OR = 0.39) also had reduced odds for low‐to‐medium BAC. The time since the last visit to healthcare services was strongly associated with higher odds of both low‐to‐medium and high BAC, with ORs ranging from 1.54 to 2.53 for each category greater than the reference of 1 day. The OR for high BAC was between 1.65 and 2.04 for each category up to a year, with categories beyond a year not statistically significant. Previous suicide attempts had ORs of 1.42–1.60 in low‐to‐medium BAC, and 1.53–1.65 in high BAC. Finnish citizen status was not statistically significant.

### 4.3 Medical Conditions

Alcohol use had the strongest association, with confirmed diagnosis ORs of 275.27 for high BAC and 41.80 in low‐to‐medium BAC. Schizophrenia (OR = 0.17) and bipolar disorder (OR = 0.29) had reduced odds in the high BAC group. There were no other confirmed diagnoses with statistically significant ORs in the low‐to‐medium BAC group. However, schizophrenia (OR = 0.63) and bipolar disorder (OR = 0.62) had reduced odds if only death certificate (autopsy) diagnosis was considered.

Likewise, some conditions only had a statistically significant result on either the healthcare register or the death certificate (autopsy) diagnosis category. Drug use had elevated odds but only by autopsy, with ORs of 2.19 in the high BAC and 1.95 in the low‐to‐medium BAC groups. Respiratory disease diagnosis had an OR of 0.09 on autopsy for the high BAC group, whilst circulatory diseases had ORs of 2.27 (high BAC) and 1.73 (low‐to‐medium BAC) on autopsy diagnosis. Unspecified mental health disorders had ORs of 0.25 (high BAC) and 0.48 (low‐to‐medium BAC) only on healthcare register diagnosis. More details can be found in Appendix A.

## 5 Discussion

High BAC at suicide was strongly associated with habitual alcohol use, time since last healthcare visit, previous suicide attempt(s), age, gender, schizophrenia, and bipolar disorder diagnosis.

While there are numerous studies exploring BAC levels in suicide deaths, there is limited research considering many psychiatric diagnoses and sociodemographic factors simultaneously. This study is the first to our knowledge that divides each reported disease into a more precise three categories for analysis, including register, death certificate (autopsy), and a combined diagnosis. It is also the first study in Finland that takes these variables into consideration together with sociodemographic and medical history characteristics in its analysis of suicide cases.

### 5.1 Medical History

Our findings indicate that documented habitual alcohol use was by far the most statistically significant risk factor for low‐to‐medium and high BAC at death. The significantly increased odds (ORs: 41.8–275.3) suggest individuals with high BAC at death are likely to have had previous habitual or problematic drinking habits, which may have contributed to their suicidal behaviour. Habitual alcohol consumption, including significant intoxication prior to death, could have contributed to emotional dysregulation and impulsivity, increasing the likelihood of suicidal behaviour. Conversely, it is possible that other problems, such as trauma or interpersonal conflict, which were not recorded in this study, contributed to problematic drinking in high BAC suicide deaths. Alcohol is a well‐established coping mechanism for stress and trauma [19]. In addition, Kõlves et al. [16] found that relationship or interpersonal conflict were also risk factors for suicide in BAC‐positive cases. The low‐to‐medium BAC category in our study may be insufficient to substantially influence behavioural disinhibition, compared with the high BAC group.

Among diagnosed conditions, schizophrenia and bipolar disorder were statistically significant and reduced the odds of high BAC at death. In this BAC category, the relative odds of a suicide death where a person was also diagnosed with schizophrenia or bipolar disorder were 17% and 29%, respectively. This is reflected in previous literature on the topic, which also shows that these conditions are less likely in BAC‐positive suicide cases. Several other conditions were statistically significant only in the healthcare register or autopsy categories. These included drug use and circulatory diseases, which had increased odds when considering only autopsy results. Meanwhile, respiratory diseases (autopsy) and unspecified mental disorders (healthcare register) had decreased odds. The results may indicate different underlying mechanisms for suicide in alcohol‐positive cases, particularly at high BAC levels, compared with schizophrenia and bipolar disorder, which are among the mental disorders with the highest burden of disease on society [20]. Our study results were strengthened by the division of BAC level at death to low‐to‐medium (0.010%–0.099%) and high (0.100%–0.500%).

### 5.2 Sociodemographic Factors

There were noteworthy findings regarding sociodemographic risk factors. Both the youngest age group (10–19 years) and the highest age groups (70–79 and 80+ years) had decreased odds for an elevated BAC at suicide. This is consistent with previous research and may reflect age‐specific alcohol consumption patterns. Recent studies including Kraus et al. [21] have found that contemporary youth generations under 20 have lower alcohol consumption than in previous decades, with a downward trend. Drinking trends among adults aged 70 years and older are less clear, but the decreased odds of high BAC at suicide suggest that many suicide deaths among the oldest age groups have different contextual risk factors than those in the highest‐risk middle age groups.

Another key finding with limited previous research was the relationship between BAC level at suicide and the time since the last healthcare visit. Having a greater duration since the last visit generally increased the odds of a low‐to‐medium or high BAC at suicide, especially when the interval was between 1–6 months and 6–12 months. Longer intervals since healthcare contact may indicate a disengagement from services, which could contribute to escalating alcohol use, negative psychosocial symptoms, and suicidal behaviour, which can worsen in a detrimental cycle the longer the care interval is. Roos af Hjelmsäter [22] found that 55% of Swedish suicide cases in 2015 had deficiencies in healthcare prior to suicide. In combination with our findings this suggests the importance of adequate, precise and timely care available to and encouraged for at‐risk patients.

Our study found that previous suicide attempt(s) also increased the odds of an elevated BAC at suicide by 42%–65%, indicating that these cases were more likely to have been involved with suicidal behaviour longer‐term. It cannot be ascertained that this behaviour was due to the alcohol or other extraneous or mediating variables such as personal circumstances, but alcohol use appears to be prevalent in cases that have had multiple suicide attempts prior to suicide. Previous suicide attempts are also the leading risk factor for suicide [7].

Our findings show that female gender had higher odds for high BAC at suicide compared to male; however, this was not statistically significant at low‐to‐medium BAC. While males generally have a higher risk for suicide, high alcohol consumption, and other alcohol use disorders, women have been found in studies such as Kaplan et al. [23] to have heightened odds (OR = 6.2) of death by suicide while intoxicated. This could be explained by the lower biological threshold for similar volumes of alcohol to induce high BAC levels in women, therefore influencing risk‐taking behaviour, although the finding warrants further investigation.

## 6 Strengths and Limitations

The main strengths of our study include its significant population‐based sample, broad range of conditions examined, objective reporting, and precision of diagnosis on three levels: death certificate (autopsy), healthcare register, and a combined measure. Furthermore, the size of the population and presence of numerous sociodemographic measures allowed for meticulous statistical analysis on the association of each measure with BAC in suicide deaths. This was partly due to each category and variable having relatively high sample sizes. The data on previous healthcare visits and number of suicide attempts also strengthened our analysis in determining the relationship these factors had on BAC levels in suicide deaths. Being able to categorise the BAC groups into low‐to‐medium and high added further precision to our analysis in ascertaining dose‐dependent effects. It allowed a more focused approach to the variables influencing the three groups: nil, low‐to‐medium, and high BAC.

Several limitations exist in our work. Notably, the lack of a control group of non‐suicide patients prevents a more robust analysis of factors and comparison between suicidal and non‐suicidal groups. The study was not able to establish the method of suicide, which has shown in some studies [24] to be associated with BAC levels, specifically that higher lethality methods are more prevalent at high BAC levels. It is also a limitation that some of the cases with a very low positive BAC of 0.010% (0.1 g/L) may have yielded this result due to decomposition of the body, where postmortem endogenous alcohol production, if it occurred, may have hampered accurate assessment of BAC in victims of fatal injuries, thereby increasing the number of suicide deaths reported in the low‐to‐medium BAC category. These cases were not removed from the dataset. Furthermore, the absence of data for other suicide risk factors such as interpersonal conflict, relationship breakdown, or trauma, which have been noted in previous studies [16] to have statistically significant relationships with BAC levels at death, represent a key drawback. Our study also did not record some sociodemographic data of interest, such as income, socioeconomic status, household composition, education, marital status, and criminal record, which are generally considered to have a relationship with suicide risk, although the literature on their relationship with BAC levels at suicide is still limited. The data was only collected from the Finnish population, so generalising the findings to a global context may be difficult to justify until a larger body of literature within this focus area is established.

### 6.1 Further Research

Future studies in this field should consider control groups and continue to incorporate a robust sample size, precise diagnostic data, accurate toxicology reports, along with a greater emphasis on sociodemographic factors and suicide methods. Comparing BAC levels among a group of suicide deaths and unsuccessful suicide attempts could be examined.

### 6.2 Clinical and Suicide Prevention Interventions

Our findings have several implications for clinical practice and suicide prevention strategies globally. Acute alcohol intoxication appears to function as a proximal risk amplifier in suicide. This is more pronounced among individuals with documented habitual alcohol use and previous suicide attempts. Alcohol use, suicidal behaviour and psychiatric symptoms should be systematically assessed in suicide risk evaluations in primary and psychiatric care settings. At emergency department visits, individuals presenting with a high BAC should be considered to be in a critical intervention window, during which structured suicide screening and brief alcohol interventions would already be beneficial. Subsequently, individuals should be followed up at regular, proactive intervals in a systematic fashion. Emerging technologies could assist in monitoring at‐risk individuals and prompting follow‐up.

The association between high BAC and longer healthcare contact intervals indicates that individuals disengaged from services may be at higher risk, and hence they are a clinically vulnerable subgroup. Any patients with a history of suicide attempts and habitual alcohol usage without recent healthcare contact should be a priority group for proactive outreach strategies.

Our finding that women had increased odds of high BAC at suicide emphasises that alcohol‐related suicide risk among women is significant. Although men account for the majority of suicide deaths, women presenting to healthcare services with acute or habitual alcohol use along with psychiatric symptoms or a history of self‐harm should be carefully assessed for suicide risk.

Overall, improving alcohol use screening, monitoring healthcare engagement, and structured follow‐up for individuals with prior suicide attempts may enhance suicide prevention efforts within both primary and specialised healthcare settings.

## 7 Conclusion

Our study found that documented habitual alcohol use, female gender, previous suicide attempts, and a longer time since the last healthcare visit increased the odds of a high BAC (0.10%, that is 1.00 g/L, or greater) at suicide. It also found that young age (below 20 years) and older age (70 years and above) or being diagnosed with schizophrenia or bipolar disorder decreased the odds of having a high BAC at suicide. The results emphasise the need for suicide prevention strategies addressing higher risk groups, incorporating systematic alcohol‐use assessment, attention to healthcare disengagement, and structured follow‐up of individuals with prior suicide attempts. Overall, our study provides detailed population‐level evidence regarding factors associated with elevated BAC‐risk in suicide deaths in Finland.

## Author Contributions

**Tristan Pokornyi:** conceptualisation, formal analysis, investigation, methodology, software, validation, visualisation, writing – original draft, writing – review and editing. **Marjut Grainger:** data curation, investigation, resources, software. **Timo Partonen:** conceptualisation, data curation, formal analysis, investigation, methodology, project administration, resources, supervision, validation, writing – original draft, writing – review and editing.

## Funding

The authors have nothing to report.

## Ethics Statement

The authors assert that all the procedures contributing to this work comply with the ethical standards of the relevant national and institutional committees on human experimentation, as well as with the Declaration of Helsinki as adopted by the World Medical Association in 1964 and amended thereafter. All the procedures involving human participants were approved (Dnro THL/2010/6.02.00/2018) by the institutional review board (IRB 00007085) of THL (FWA 00014588) on 20 November 2018.

## Conflicts of Interest

The authors declare no conflicts of interest.

## Data Availability

The data that support the findings of this study are available from the Finnish Institute for Health and Welfare (THL) upon reasonable request due to ethical and privacy considerations, and subject to permission and data access regulations.

## Appendix A

See Tables A1, A2, A3, A4, A5.

**TABLE A1:** Year of death.

		Frequency	Percent	Cumulative Percent
Valid	2016	792	11.5	11.5
	2017	826	12.0	23.5
	2018	806	11.7	35.2
	2019	742	10.8	45.9
	2020	722	10.5	56.4
	2021	747	10.8	67.3
	2022	746	10.8	78.1
	2023	756	11.0	89.0
	2024	755	11.0	100.0
	Total	6892	100.0	

**TABLE A2:** Blood alcohol concentration (BAC) categories.

		Valid *N*	Percent
Valid	Nil	4380	63.6
	0.01–0.05 BAC	419	6.1
	0.05–0.10 BAC	508	7.4
	0.10–0.15 BAC	510	7.4
	0.15–0.20 BAC	494	7.2
	0.20–0.25 BAC	327	4.7
	0.25–0.30 BAC	132	1.9
	> 0.30 BAC	65	0.9
	Total	6835	99.2
Missing	System	57	0.8
Total		6892	100.0

**TABLE A3:** Gender N/M × alcohol concentration three categories cross‐tabulation.

		Alcohol concentration three categories			Total
		Nil BAC 0.00	Low–medium BAC 0.01–0.09	High BAC 0.10–0.50	
Gender N/M					
Male	Count	3200	715	1228	5143
	% within gender F/M	62.2%	13.9%	23.9%	100.0%
	% within alcohol concentration three categories	73.1%	77.1%	80.4%	75.2%
	% of total	46.8%	10.5%	18.0%	75.2%
Female	Count	1180	212	300	1692
	% within gender F/M	69.7%	12.5%	17.7%	100.0%
	% within alcohol concentration three categories	26.9%	22.9%	19.6%	24.8%
	% of total	17.3%	3.1%	4.4%	24.8%
Total	Count	4380	927	1528	6835
	% within gender F/M	64.1%	13.6%	22.4%	100.0%
	% within alcohol concentration three categories	100.0%	100.0%	100.0%	100.0%
	% of total	64.1%	13.6%	22.4%	100.0%

**TABLE A4:** Wellbeing services county name.

	Valid *N*	Percent
Not stated	90	1.3
Ahvenanmaa	29	0.4
Etelä‐Karjalan hyvinvointialue	155	2.2
Etelä‐Pohjanmaan hyvinvointialue	217	3.1
Etelä‐Savon hyvinvointialue	214	3.1
Helsingin kaupunki	685	9.9
Itä‐Uudenmaan hyvinvointialue	95	1.4
Kainuun hyvinvointialue	124	1.8
Kanta‐Hämeen hyvinvointialue	242	3.5
Keski‐Pohjanmaan hyvinvointialue	64	0.9
Keski‐Suomen hyvinvointialue	374	5.4
Keski‐Uudenmaan hyvinvointialue	243	3.5
Kymenlaakson hyvinvointialue	211	3.1
Länsi‐Uudenmaan hyvinvointialue	470	6.8
Lapin hyvinvointialue	244	3.5
Päijät‐Hämeen hyvinvointialue	282	4.1
Pirkanmaan hyvinvointialue	613	8.9
Pohjanmaan hyvinvointialue	198	2.9
Pohjois‐Karjalan hyvinvointialue	217	3.1
Pohjois‐Pohjanmaan hyvinvointialue	577	8.4
Pohjois‐Savon hyvinvointialue	379	5.5
Satakunnan hyvinvointialue	286	4.1
Vantaan ja Keravan hyvinvointialue	288	4.2
Varsinais‐Suomen hyvinvointialue	595	8.6
Total	6892	100.0

**TABLE A5:** Logistic regression—all diagnostic levels included.

Alcohol concentration three categories^a^—nil BAC is reference category	Sig.	Exp(*B*)	95% confidence interval for Exp(*B*)	
			Lower bound	Upper bound
*Low–medium BAC 0.010%–0.099%*				
Gender: female	0.945	1.007	0.833	1.217
Gender: male (REF)				
Age at death: 10–19	0.047	0.647	0.421	0.994
Age at death: 20–29	0.699	1.055	0.804	1.384
Age at death: 30–39	0.962	1.007	0.765	1.324
Age at death: 40–49 (REF)				
Age at death: 50–59	0.749	1.047	0.791	1.385
Age at death: 60–69	0.327	0.863	0.642	1.159
Age at death: 70–79	0.381	0.864	0.623	1.199
Age at death: 80 and above	< 0.001	0.390	0.241	0.631
Earlier attempts = 1	0.021	1.422	1.055	1.917
Earlier attempts = 2 or more	< 0.001	1.604	1.283	2.005
Earlier attempts = none (REF)				
Last visit to healthcare = 1–7 days ago	0.001	1.540	1.205	1.968
Last visit to healthcare = more than a week but less than a month	< 0.001	2.176	1.692	2.799
Last visit to healthcare = 1–6 months ago	< 0.001	2.254	1.735	2.930
Last visit to healthcare = 6–12 months ago	0.001	2.009	1.340	3.014
Last visit to healthcare = 1–2 years ago	0.008	1.862	1.179	2.942
Last visit to healthcare = More than 2 years ago	< 0.001	2.529	1.625	3.936
Last visit to healthcare = within past day (REF)				
Citizenship = foreigner	0.169	0.344	0.075	1.576
Citizenship = Finnish citizen (REF)				
Cancer diagnosis = healthcare register only	0.103	1.186	0.966	1.455
Cancer diagnosis = death certificate/autopsy only	0.684	1.183	0.526	2.659
Cancer diagnosis = both	0.667	1.360	0.335	5.529
Cancer diagnosis = none (REF)				
Alcoholic disease diagnosis = healthcare register only	0.154	1.341	0.896	2.008
Alcoholic disease diagnosis = death certificate/autopsy only	0.346	0.578	0.185	1.808
Alcoholic disease diagnosis = both	0.264	3.417	0.396	29.456
Alcoholic disease diagnosis = none (REF)				
Alcohol use = healthcare register only	0.029	1.370	1.033	1.817
Alcohol use = death certificate/autopsy only	< 0.001	24.856	19.231	32.126
Alcohol use = both	< 0.001	41.799	19.361	90.242
Alcohol use = none (REF)				
Drug use = healthcare register only	0.212	1.247	0.881	1.765
Drug use = death certificate/autopsy only	< 0.001	1.950	1.494	2.545
Drug use = both	0.079	1.611	0.946	2.744
Drug use = none (REF)				
Schizophrenia = healthcare register only	0.468	0.880	0.624	1.242
Schizophrenia = death certificate/autopsy only	0.018	0.625	0.424	0.921
Schizophrenia = both	0.872	1.064	0.501	2.262
Schizophrenia = none (REF)				
Bipolar = healthcare register only	0.051	1.657	0.997	2.755
Bipolar = death certificate/autopsy only	0.045	0.621	0.390	0.989
Bipolar = both	0.563	0.755	0.292	1.955
Bipolar = none (REF)				
Depression = healthcare register only	0.606	1.083	0.801	1.465
Depression = death certificate/autopsy only	0.099	1.180	0.969	1.438
Depression = both	0.323	1.197	0.838	1.711
Depression = none (REF)				
Respiratory disease diagnosis = healthcare register only	0.357	0.914	0.756	1.106
Respiratory disease diagnosis = death certificate/autopsy only	0.304	0.553	0.179	1.711
Respiratory disease diagnosis = both	^b^	^b^	^b^	^b^
Respiratory disease diagnosis = none (REF)				
Circulatory diseases diagnosis = healthcare register only	0.587	0.938	0.744	1.182
Circulatory diseases diagnosis = death certificate/autopsy only	0.022	1.725	1.082	2.752
Circulatory diseases diagnosis = both	0.526	1.401	0.494	3.972
Circulatory diseases diagnosis = none (REF)				
Unspecified mental health disorder diagnosis = healthcare register only	0.002	0.481	0.305	0.757
Unspecified mental health disorder diagnosis = death certificate/autopsy only	0.377	1.669	0.536	5.199
Unspecified mental health disorder diagnosis = none (REF)				
*High BAC 0.100%–0.500%*				
Gender: female	0.017	1.297	1.047	1.605
Gender: male (REF)				
Age at death: 10–19	< 0.001	0.306	0.185	0.507
Age at death: 20–29	0.235	0.835	0.620	1.124
Age at death: 30–39	0.519	0.907	0.674	1.220
Age at death: 40–49 (REF)				
Age at death: 50–59	0.282	1.173	0.877	1.570
Age at death: 60–69	0.079	0.753	0.550	1.033
Age at death: 70–79	< 0.001	0.427	0.289	0.631
Age at death: 80 and above	< 0.001	0.145	0.074	0.283
Earlier attempts = 1	0.002	1.650	1.196	2.278
Earlier attempts = 2 or more	0.001	1.526	1.184	1.967
Earlier attempts = none (REF)				
Last visit to healthcare = 1–7 days ago	0.177	1.204	0.920	1.577
Last visit to healthcare = more than a week but less than a month	< 0.001	1.646	1.250	2.167
Last visit to healthcare = 1–6 months ago	< 0.001	2.041	1.548	2.691
Last visit to healthcare = 6–12 months ago	0.021	1.665	1.079	2.567
Last visit to healthcare = 1–2 years ago	0.141	1.443	0.886	2.351
Last visit to healthcare = More than 2 years ago	0.382	1.255	0.755	2.085
Last visit to healthcare = within past day (REF)				
Citizenship = foreigner	0.880	1.095	0.336	3.574
Citizenship = Finnish citizen (REF)				
Cancer diagnosis = healthcare register only	0.131	1.194	0.949	1.502
Cancer diagnosis = death certificate/autopsy only	0.119	0.357	0.098	1.304
Cancer diagnosis = both	0.320	0.283	0.023	3.410
Cancer diagnosis = none (REF)				
Alcoholic disease diagnosis = healthcare register only	0.134	1.397	0.902	2.165
Alcoholic disease diagnosis = death certificate/autopsy only	0.847	0.904	0.326	2.509
Alcoholic disease diagnosis = both	0.355	2.867	0.308	26.723
Alcoholic disease diagnosis = none (REF)				
Alcohol use = healthcare register only	0.003	1.645	1.182	2.288
Alcohol use = death certificate/autopsy only	< 0.001	148.594	115.111	191.817
Alcohol use = both	< 0.001	275.270	131.999	574.045
Alcohol use = none (REF)				
Drug use = healthcare register only	0.910	0.977	0.654	1.461
Drug use = death certificate/autopsy only	< 0.001	2.194	1.625	2.963
Drug use = both	0.945	1.025	0.505	2.081
Drug use = none (REF)				
Schizophrenia = healthcare register only	0.064	0.683	0.456	1.022
Schizophrenia = death certificate/autopsy only	< 0.001	0.248	0.142	0.433
Schizophrenia = both	0.013	0.168	0.041	0.690
Schizophrenia = none (REF)				
Bipolar = healthcare register only	0.886	0.952	0.490	1.853
Bipolar = death certificate/autopsy only	0.065	0.624	0.378	1.030
Bipolar = both	0.040	0.285	0.086	0.943
Bipolar = none (REF)				
Depression = healthcare register only	0.890	1.024	0.729	1.440
Depression = death certificate/autopsy only	0.670	1.049	0.842	1.306
Depression = both	0.856	1.038	0.692	1.559
Depression = none (REF)				
Respiratory disease diagnosis = healthcare register only	0.115	0.842	0.681	1.042
Respiratory disease diagnosis = death certificate/autopsy only	0.007	0.093	0.017	0.517
Respiratory disease diagnosis = both	^b^	^b^	^b^	^b^
Respiratory disease diagnosis = none (REF)				
Circulatory diseases diagnosis = healthcare register only	0.172	0.832	0.639	1.083
Circulatory diseases diagnosis = death certificate/autopsy only	0.002	2.266	1.365	3.763
Circulatory diseases diagnosis = both	0.622	1.335	0.423	4.214
Circulatory diseases diagnosis = none (REF)				
Unspecified mental health disorder diagnosis = healthcare register only	< 0.001	0.246	0.144	0.423
Unspecified mental health disorder diagnosis = death certificate/autopsy only	0.432	1.684	0.459	6.182
Unspecified mental health disorder diagnosis = none				

^a^ The reference category is: NIL BAC 0.00.

^b^ Not estimable—predictors with sparse data or unstable estimates were excluded from the main results table (see appendices for full output).
